# Effects of Central and Peripheral Fatigue on Impact Characteristics during Running

**DOI:** 10.3390/s22103786

**Published:** 2022-05-16

**Authors:** Alberto Encarnación-Martínez, Antonio García-Gallart, Roberto Sanchis-Sanchis, Pedro Pérez-Soriano

**Affiliations:** 1 Research Group in Sports Biomechanics (GIBD), Department of Physical Education and Sports, University of Valencia, 46010 Valencia, Spain; roberto.sanchis@uv.es (R.S.-S.); pedro.perez-soriano@uv.es (P.P.-S.); 2 Department of Sports Sciences, Universidad Católica de Murcia UCAM, 30107 Murcia, Spain; 3 The Civil Guard, Secretary of State for Security, Ministry of the Interior, 28010 Madrid, Spain; garciagallart@gmail.com

**Keywords:** impacts, peripheral, central, fatigue, running

## Abstract

Fatigue and impact can represent an injury risk factor during running. The objective of this study was to compare the impact transmission along the locomotor system between the central and peripheral fatigued states during running. Tibial and head acceleration as well as shock attenuation in the time- and frequency-domain were analyzed during 2-min of treadmill running in the pre- and post-fatigue state in eighteen male popular runners (N = 18). The impact transmission was measured before and after a 30-min central fatigue protocol on the treadmill or a peripheral fatigue protocol in the quadricep and hamstring muscles using an isokinetic dynamometer. The time-domain acceleration variables were not modified either by peripheral or central fatigue (*p* > 0.05). Nevertheless, central fatigue increased the maximum (*p* = 0.006) and total (*p* = 0.007) signal power magnitude in the high-frequency range in the tibia, and the attenuation variable in the low- (*p* = 0.048) and high-frequency area (*p* = 0.000), while peripheral fatigue did not cause any modifications in the frequency-domain variables (*p* > 0.05). Furthermore, the attenuation in the low (*p* = 0.000)- and high-frequency area was higher with central fatigue than peripheral fatigue (*p* = 0.003). The results demonstrate that central fatigue increases the severity of impact during running as well as the attenuation of low and high components.

## 1. Introduction

Running is one of the most practiced physical activities in the world, but almost half of these runners are injured every year [1]. With each foot contact with the ground, an impact wave is propagated along the locomotor system, which stresses up to 1.5 to 2.5 times the body [2,3,4], and it is absorbed by the whole body from the foot to the head [5,6,7]. Even so, the running impact is very high and it could be related to stress injuries [2,3,4,7,8,9].

Some factors could increase the severity of the impacts. One of them is fatigue [3,9,10,11], which can be defined as a multifactorial response related to exercise that is characterized by decreasing muscle strength or power [12]. Fatigue has been traditionally divided into peripheral fatigue and central fatigue. Peripheral fatigue generates modifications at the muscular level, hindering the execution of central commands while central fatigue is produced by limitations at the spinal or supraspinal level [12].

Several studies have investigated the effects of central fatigue on the running impacts [3,7,9,11,13,14,15]. Conversely, the investigations that have analyzed the influence of peripheral fatigue in specific muscle groups on the impact transmission during running are limited [16]. In addition, there have been no studies that have compared the effects of both types of fatigue evaluated in isolation under the same methodology in running impacts.

Recent studies have shown that there is great heterogeneity in the protocols used to describe the effects of fatigue on the acceleration impacts [17], describing the fatigue protocols that range from 13.6 to 48.5 min in duration [17]. These studies have shown that fatigue significantly increases the impact accelerations in 46% of running studies. The differences found in the results could be due to the duration and characteristics of the fatigue protocols, which do not respond to the characteristics of the internal processes (energy and neuromuscular) that modulate the appearance of fatigue.

Traditionally, impacts have been evaluated in the time domain analysis [9,11,13,14]. However, their analysis in the frequency-domain allowed for a more detailed study since it could analyze the frequency components of the signal, directly determining the transmissibility of the impact on the human body [6,18]. Spectral analysis or frequency-domain analysis is a method that, together with the application of appropriate mathematical procedures, allows one to separate the different components of the frequencies produced during the impact acceleration to distinguish the different components of acceleration from low-frequency movements (characteristic of human movement) and high-frequency resonance (related to the severity of the impacts) in the accelerometer instrumentation [18].

In contrast, to the authors’ knowledge, the studies that have evaluated the effect of fatigue on the running impacts under this frequency-domain are few in number [2,3,7,15]. Therefore, the main objective of this study was to compare the impact transmission between the central and peripheral fatigued states during running under the same methodology and from the time- and frequency-domain perspectives.

The key contributions of this paper can be summarized as follows:

- We compared the impact acceleration response to fatigue under two well-stabilized protocols to allow us to understand the response after the central and peripheral fatigue situations.
- We compared the two methods to analyze the impact acceleration, time, and frequency analysis, which showed that the frequency-domain was the best option to detect fatigue-related changes.
- We present here that central fatigue increases the severity of impact acceleration, and propose to include these kinds of protocols in future studies focus on the identification of factors related to running-related injuries (RRIs).

## 2. Materials and Methods

### 2.1. Participants

Eighteen male recreational runners with an average age of 28.2 ± 8.6 years, height of 1.77 ± 0.65 m, body mass of 71.74 ± 8.44 kg, the estimated maximal oxygen consumption (VO_2_max) of 62.2 ± 4.7 mL/kg/min, and experience of 7.3 ± 5.3 years participated in the study. All subjects were required to run a minimum of twice a week in the last year and not be injured in the previous six months. All participants had previous treadmill running experience. The final sample size was previously calculated using the G-Power 3 software (version 3.1.9.7, Düsseldorf, Germany). The results indicated that at least a sample of 12 recreational runners was required to detect statistical differences in the variables with a minimum detectable effect size of f = 0.8 (large) (α = 0.05, β = 0.05, power = 0.96).

The University Ethics Committee approved this investigation (registry number: 6775) and all participants provided informed consent before inclusion in the study.

### 2.2. Experimental Protocol

**Figure 1:**
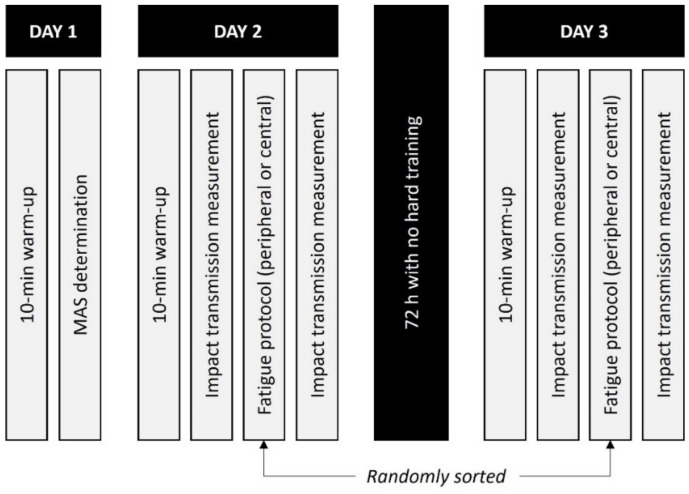
The experimental protocol followed in the study.

The study was carried out on three separate days (Figure 1). On the first day, participants performed a maximal effort 5-min running test on a 400-m track, which was used in multiple investigations as a measurement of maximal aerobic speed (MAS) [11,13,19]. The speed at which VO_2_max is reached was calculated indirectly. The distance reached during the 5-min test at the highest sustained speed was multiplied by 12, obtaining the MAS in km/h, known as the lowest speed requested by VO_2_max [18]. The MAS was used to individualize the speed of the central fatigue test, for which we selected 85% of the MAS. The mean and standard deviation of the MAS was 4.9 ± 0.4 m/s, indicating a good level of the athletes participating in the study. Then, the mean speed at 85% of MAS was set at 4.2 ± 0.3 m/s.

Impact transmission was measured on the second and third days. In every session, the participants performed a 10-min warm-up at a self-selected speed on a treadmill (Excite^®^+ Run MD Inclusive, Technogym Trading S.A., Barcelona, Spain) [11,13]. Then, the participants ran for 2-min at 3.89 m/s [20] and 0% slope, in order not to affect the parameters evaluated [21]. 

Impact transmission during running was recorded in the last 30 s of this 2-min period through three series of 10 s to ensure the adaptation to running on the treadmill and to the speed of the test. A wireless triaxial accelerometry was used (Blautic^®^, Valencia, Spain) with a sampling frequency of 300 Hz, a measuring range of up to 16 g, and a mass of 2.5 g. The low-mass accelerometers employed in the study, together with the data processing, minimize the artifact movement interferences [22].

Because the location of the tibial accelerometer does influence the acceleration signal [23], one accelerometer was placed in the distal and anteromedial portion of the tibia [23] in the dominant leg and other accelerometer was placed in front of the head [3,5,6,7,11,13,18]. Previously, the skin was prepared and the accelerometers were fixed as recommended by Encarnación-Martínez et al. [5].

Once the pre-fatigue test was finished, the participants performed a peripheral or central fatigue protocol, and then they repeated the 2-min running test. The transition between fatigue protocols and the post-fatigue running test was conducted as quickly as possible to avoid recovery processes, always before 5 min after the fatigue test [24]. Central and peripheral fatigue protocols were randomized and separated by a minimum of 72 h.

#### 2.2.1. Central Fatigue Protocol

**Figure 2:**
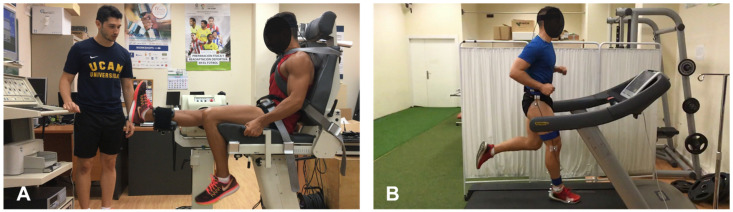
Setup of the peripheral fatigue protocol (**A**) and the central fatigue protocol (**B**).

To induce central fatigue, the participants ran for 30-min on a treadmill at 85% MAS (Figure 2B) [13], determined on the first day in the 5-min running field test, and the 0% slope was adjusted on the treadmill [21]. Additionally, to ensure that the participants reached an adequate level of fatigue, they must manifest a perceived effort of 17 or “Very Hard” [25] on the Borg’s Scale 6–20 [26]. A recent systematic review that analyzed the effects of running fatigue on the impact acceleration reported a mean duration of protocols of about 28.8 min, ranging from 13.6 to 48.5 min, with the present study in line with previous studies [17]. 

#### 2.2.2. Peripheral Fatigue Protocol

The peak concentric torque and peripheral fatigue protocols were carried out in the quadricep and hamstring muscles of the dominant lower extremity using an isokinetic dynamometer (Biodex System Pro 3™, Biodex Medical Systems, Inc., New York, NY, USA). Both tests were performed in a seated position with a hip flexion angle of 85°, and with the trunk, waist, and thigh of the domain lower extremity stabilized with straps [27,28].

First, the peak torque values were evaluated by performing two sets of concentric/concentric knee flexion–extension movements at 120°/s (Figure 2A). The motion ranged from 0° (full extension) to 90° of knee flexion [27,28]. The first set was conducted as familiarization and consisted of three submaximal and three maximal contractions [28]. In the second set, the concentric peak torque of the quadricep and hamstring muscles was registered, performing three repetitions of maximal effort through the whole range of motion within rest [27,28]. The highest quadricep and hamstring peak torque of three repetitions was recorded as the concentric peak torque [27,28]. 

After a 3-min rest period, the peripheral fatigue protocol was performed [28]. Participants were instructed to perform continuous concentric/concentric knee flexion–extension movements at 120°/s, exerting the maximal effort through the whole range of motion within rest. The peripheral fatigue threshold was set when the concentric peak torque fell below 50% for three consecutive movements in both directions [28]. The researcher who measured this protocol provided verbal cheering to the participants to induce proper peripheral fatigue.

### 2.3. Data Analysis

A custom routine performed with the MATLAB R2013b program (Mathworks Inc, Natick, MA, USA) was used to analyze the acceleration data. In the time-domain analysis, a low pass Butterworth filter with a cutoff frequency of 50 Hz was applied [5,6]. The peak acceleration of tibia (PAT), the peak acceleration of head (PAH), and the tibia and head ratios of acceleration (RAT and RAH, respectively) were obtained and measured in gravity (g) (1 g = 9.82 m/s^2^). Therefore, the peak acceleration describes the maximum amplitude of acceleration during the initial contact [13,15], while the ratio of acceleration represents the differences between the positive and the negative peak [11]. The higher the values, the higher the severity of the impact.

Moreover, the magnitude of impact absorption (MIA) was calculated by the percentage of the tibia peak acceleration absorbed by the body when reaching the head [5,7,11,13] (Equation (1)):

MIA (%) = 100 − [(PAH/PAT) × 100](1)

The stride frequency and stride length were also calculated. The time (s) between the two consecutive tibial acceleration peaks was used to detect the stride frequency (Hz), and the treadmill speed (m/s) was divided by the stride frequency to obtain the stride length (m) [29].

On the other hand, the unfiltered time-domain data were converted to the frequency domain parameters and power spectral density (PSD) as described by Gruber et al. [6]. Later, the maximum and total signal power magnitude in the low (3–8 Hz)- and high (9–20 Hz)-frequency range in the tibia (MTSM_low_, MTSM_high_, TTSM_low_, and TTSM_high_, respectively) and head were obtained (MHSM_low_, MHSM_high_, THSM_low_, and THSM_high_, respectively) [6].

The low-frequencies (3–8 Hz) are related to the cyclical movements of the human being (the movement), while high frequencies (9–20 Hz) are related to the intensity or magnitude of the impact against the ground. Higher values at the maximum or total signal represent, in the low zone, an increase in movement; whereas in the high zone, it represents an increase in the severity of the impact [6].

Moreover, the shock attenuation (ATT) was calculated in the low- and high-frequency areas (ATT_low_ and ATT_high_, respectively) by the following equation [3,6,15,18] (Equation (2)):

Shock Attenuation = 10 × log10 (PSDhead/PSDtibia)(2)

The positive values in the Shoch attenuation indicate an increase in the signal strength from the tibia to the head, while the negative values suggest an attenuation of the signal strength.

Pre-post changes (Δ) after peripheral and central fatigue were calculated in all of the time- and frequency-domain variables.

### 2.4. Statistics

Descriptive statistics are described as the means ± standard deviation (SD) and the Shapiro–Wilk test and Levene test were used to examine the normality of the data distribution and the homoscedasticity of the variances, respectively. A two-way repeated measures ANOVA or its non-parametric alternative, the Friedman test, was used to compare the impact characteristics between the pre- and post-fatigue conditions and peripheral and central fatigue. The Bonferroni or Wilcoxon post hoc test was performed to identify the location of specific differences. The paired samples t-test or Wilcoxon test were used to calculate the differences between the delta (Δ) changes in the peripheral and central fatigue running protocols. Afterward, the confidence intervals of the differences (95% CI) and effect sizes (ES) were calculated to identify meaningful changes. The effect size (ES) was analyzed using Cohen´s d [30] through the formula proposed by Hunter and Schmidt [31], and it was interpreted as: 0.0–0.2, very small; 0.2–0.5, small; 0.5–0.8, medium; 0.8–1.2, large; 1.2–2.0, very large; and >2.0, huge [32].

## 3. Results

Eighteen participants (N = 18) completed the peripheral fatigue protocol and seventeen runners (N = 17) finished the central fatigue protocol because a runner was injured before performing this protocol and was excluded. The average quadricep and hamstring concentric peak torque was 242.7 ± 39.9 Nm·BW^−1^ and 125.4 ± 30.5 Nm·BW^−1^, respectively. After the peripheral fatigue protocol, the level of fatigue for the quadricep and hamstring muscles was 55.8 ± 5.8% and 53.5 ± 6.4%, respectively. A mean of 38.8 ± 6.9 repetitions was needed to ensure peripheral fatigue. Otherwise, all participants completed the 30-min stipulated in the central fatigue protocol where the average running velocity was 4.2 ± 0.3 m·s^−1^ and the final perceived effort was 17.6 ± 0.5.

The time domain acceleration variables (PAT, PAH, and MAI) (Table 1), the stride frequency, and the stride length (Table 2) were not modified with either the peripheral or central fatigue (*p* > 0.05). 

However, when analyzing between the pre- and post-fatigue conditions in the frequency-domain analysis, the central fatigue increased the maximum (MTSM_high_) (ES = 0.595, *p* = 0.006) and total (TTSM_high_) (ES = 0.488, *p* = 0.007) signal power magnitude in the high-frequency range in the tibia location, and the attenuation in low- (ATT_low_) (ES = −0.283, *p* = 0.048) and high-frequency area (ATT_high_) (ES = −1.396, *p* = 0.000). Instead, peripheral fatigue did not cause modifications in the tibial and head accelerations and shock attenuation in the frequency domain (*p* > 0.05) (Table 2). In addition, when comparing the fatigue protocols, ATT_low_ (ES = −0.541, *p* = 0.000) and ATT_high_ (ES = −0.896, *p* = 0.003) were higher with central fatigue than peripheral fatigue, and the post-fatigue increase was significantly greater in the central fatigue than the peripheral fatigue in ΔMTSM_high_ (ES = 0.885, *p* = 0.043), ΔTTSM_high_ (ES = 0.817, *p* = 0.029), ΔATT_low_ (ES = 0.745, *p* = 0.006), and ΔATT_high_ (95% CI = −11.543/−2.240, ES = 1.146, *p* = 0.049) (Table 3).

## 4. Discussion

The aim of this study was to identify and compare the effects of central and peripheral fatigue in impact transmission during running. Time- and frequency-domain analysis of the acceleration signal was applied to extract the variables of interest. 

There is no consensus about the effects of fatigue on the tibial acceleration during running. Some studies have suggested an increase in the tibial acceleration in the fatigue state [3,9,10,11], but not all showed this behavior [2,7]. Instead, it seems accepted that head accelerations, both in the time- [2,3,7,11,13] and frequency [3,7]-domain analyses, did not increase during fatigued running. This is consistent with the results of our study since the central and peripheral fatigue did not modify the severity of the head impact in the time- and frequency-domain analysis. It has been suggested as a protective behavior to prevent a possible disruption of the visual and vestibular system, which could occur due to excessive head acceleration [6,11]. 

In our study, central fatigue did not increase the tibial acceleration and the impact attenuation in the time-domain analysis. The different behaviors of the tibial acceleration in the fatigue state found in previous studies could be explained by the lack of consensus on the right tibia accelerometer location. Similar studies located the tibial accelerometer in the proximal part [2,9,11,13,14] while only two studies analyzed the accelerations in the distal portion of the tibia [3,7] such as in the present study.

On one hand, almost all of the studies that analyzed the proximal tibial accelerations in the time-domain indicated an increase in the tibial acceleration and shock attenuation with central fatigue [9,10,11], where the peak tibial acceleration could increase up to 50% after running for 30-min at the anaerobic threshold [9]. Nevertheless, García-Pérez et al. [13], who placed the accelerometer in the same place, reported a non-significant tibial acceleration increase; similarly, in a shorter duration protocol (17.8 ± 5.7 min), Abt et al. [2] also found no modifications in the tibia acceleration. 

On the other hand, Mercer et al. [7] placed the accelerometer in the distal portion of the tibia and did not find modifications in the acceleration after an incremental running protocol until exhaustion. Acceleration in the tibia was not altered in our study after central fatigue, coinciding with the results of Mercer et al. [7]. In opposition, in a more intense and shorter duration protocol (15.7 ± 1.7 min), Derrick et al. [3] found an increase in tibial acceleration and shock attenuation. 

Thus, in central fatigue protocols with a duration of around 30 min and intensity such as the anaerobic threshold, it seems that the tibial peak acceleration and attenuation increase in the proximal tibia accelerometer position, but these parameters are not modified in a distal placement.

It has been demonstrated that the proximal tibia accelerometer is affected by the gravity and the angular velocity of the lower leg, underestimating the axial peak acceleration in the time-domain [23]. However, the tibial accelerations in this location seem higher. This contradiction could be explained by the kinematic changes that occur at the fatigue running state. The knee angle is the kinematic variable more directly related to the impact transmission and attenuation [9]. As previously shown, with the progression of fatigue during treadmill steady-running, a gradual increase in the knee angle in the maximum extension position is generated, which is produced just preceding the foot strike position [9] and in the instant of contact [3]. This modification increases the tibial peak acceleration and impact absorption [3,4,9,33], decreases the effective mass [27], and has been described as a corporal compensatory strategy to reduce the ground reaction forces (GRF) [3,34,35,36,37]. Moreover, Potthast, Bruggemann, Lundberg, and Arndt [38] suggested that knee angle changes explained 25–29% of the variance in the proximal tibia acceleration and impact forces. Thereby, the increase in proximal tibial acceleration found in previous studies could be caused more by the increase in knee flexion and the greater angular velocity of the lower leg than by fatigue.

In the frequency-domain analysis, an increment in the high-frequency range acceleration in the tibia location and the shock attenuation with central fatigue was revealed. This analysis could underestimate the impact parameters of the low-frequency area in the proximal portion of the tibia, but the proximal or distal location of the tibial accelerometer did not alter the results of the impacts in the high-frequency range [23]. Therefore, the results obtained can be described as the body system needing to attenuate a greater amount of impact to maintain adequate impact values in the head.

Previous studies performed in level running [8,15] have described that as time passes in the 30-min running protocol, the tibial power is higher, especially in the high-frequency zone. The results of our study agree with these studies because the central fatigue increased the severity of the high-frequency impacts in the tibia location. These findings contradict those obtained in the time-domain analysis, but it should be noted that the frequency-domain analysis directly determines the transmissibility of the impact on the human body and is a more accurate analysis [18].

Low-frequency shock attenuation was more attenuated with central fatigue. Since these low-frequency accelerations represent the athlete’s body segment movements during running [4,6,18], the expected increase in the joint range of motion with central fatigue means that the body system has to further attenuate these movements to maintain constant accelerations in the head.

The high-frequency attenuation also increased with central fatigue. Mizrahi, Verbitsky et al. [8] found that attenuation between the shank and sacrum showed a major peak around 9 Hz, which increased in level running but not during decline running. Abt et al. [2] and Derrick et al. [3] found no changes in attenuation with central fatigue after an exhaustive run at the ventilatory threshold (17.8 ± 5.7 min) or average speed measured for 3.200 m (15.7 ± 1.7 min), respectively. The study by Mercer et al. [7], after a graded running protocol, showed a decrease in shock attenuation. These inconsistent results found in the literature, coinciding with Abt et al. [2], may be attributed to the different fatigue running protocols applied with different intensity, duration, or inclination. Therefore, more studies are needed to provide a clear answer as to what happens with these parameters in the fatigue state, taking into account that the shock attenuation in the frequency-domain increases as the stride frequency increases [4], and that the frequency ranges between the different footfall patterns are different and may affect how these frequencies are attenuated [4,6]. In our study, fatigue did not modify the stride frequency so the increase in attenuation was not due to an increase in stride frequency, but to an increase in the severity of impact.

Regarding peripheral fatigue, it did not increase the tibial acceleration and the impact attenuation in the time- and frequency-domain analysis. We are not aware of studies analyzing the influence of peripheral or localized fatigue on the running impacts. Christina et al. [16] examined the change in the vertical GRF in running after isokinetic fatigue in ankle dorsiflexors or foot invertors. The loading rate force increased after dorsiflexor fatigue while the impact peak magnitude and the rate of decline in the impact peak force decreased after invertor fatigue. However, these results cannot be compared with our study since other muscle groups were fatigued and the variables were not the same. 

Comparing both types of fatigue, central fatigue produced higher modifications in the tibial acceleration and shock attenuation than peripheral fatigue in the frequency-domain analysis of the impacts. As far as we know, no study has compared the effects of central and peripheral fatigue on the running impacts using a frequency-domain analysis. Only two studies have compared the central and peripheral fatigue state during running, but both types of fatigue were not induced separately, and were evaluated jointly through a voluntary activation after prolonged running [39,40]. Boccia et al. [39] described a moderate increase in central fatigue and a small one in peripheral fatigue after a half-marathon race. After a prolonged treadmill run of 24 h, Martin et al. [40] suggested that the central mechanism was mainly responsible for the reduction in the maximum knee flexor torque.

In our study, the severity of high-frequency impacts only increased with central fatigue, and both high-frequency impacts and the low- and high-frequency attenuation were higher after the central running protocol than after peripheral fatigue. The greater effects of central fatigue agree with previous studies that showed greater changes after central fatigue than peripheral fatigue during prolonged running [39,40] or cycling [41]. These higher modifications in the impact characteristics after central fatigue running protocols could be explained by the greater number of fatigued muscle groups. Kellis et al. [27] suggested that the knee, hip, and ankle muscles or joint impairments may lead to additional adjustments that would result in different muscle-activation levels compared to the localized fatigue protocols. Moreover, these running protocols reproduce, in a specific way, the sports gesture of running and produce the specific fatigue that occurs every day during training or competition. Therefore, cardiovascular, respiratory, or volitional factors are added during running fatigue protocols, which are not present in the peripheral fatigue protocols and could explain these major biomechanical modifications.

Finally, by comparing the time- and frequency-domain analysis, an increase in the severity and attenuation of impact was shown in the frequency-domain analysis during central fatigue that did not occur in the impacts evaluated by the time-domain analysis. In agreement with other authors, our findings suggest that the frequency-domain analysis is more sensitive in detecting the transmissibility of the impact on the human body during running [6,18], does not underestimate the axial peak acceleration, and is not affected by the gravity and the angular velocity of the lower leg, which happens in the time-domain analysis [23]. Futures studies should therefore use frequency-domain analysis to obtain more accurate results on the mechanical stress that the corporal system receives during running.

The present study is not without its limitations. We induced fatigue and checked their effects over the RPE estimations, as the majority of previous studies have before [17]. VO_2_max is considered as the gold standard in quantifying the internal fatigue response [42], but that technology does not allow for a comfortable and ecological situation that could affect the running performance. Future studies must include other techniques that allow us to measure other internal responses to fatigue tests. Another limitation is that our sample was composed of men, so future studies should include women in their samples to identify the sex–fatigue response.

## 5. Conclusions

An exhaustive running fatigue protocol that mainly induces central fatigue increases the severity of high-frequency impacts as well as the attenuation of low and high components. Peripheral fatigue does not modify the accelerations in the frequency-domain analysis, showing that central fatigue causes greater effects on the running impacts than peripheral fatigue. On the other hand, the time-domain accelerations remain constant after central or peripheral fatigue. This corroborates the finding that the analysis of impacts in the frequency-domain is more sensitive in assessing the severity of impacts during running. As a practical application, future studies that analyze the fatigue effects over the running biomechanics should consider that central fatigue increases the severity of the impact acceleration, therefore proposing the inclusion of long-duration protocols of high intensity running, and to relate other factors such as internal response, kinematics, or kinetic variables to identify causes of RRI.

## Figures and Tables

**Table 1:** Results (mean and standard deviation) of the impacts in the time-domain analysis, pre- and post-fatigue.

	Peripheral Fatigue			Central Fatigue		
	Pre-Fatigue	Post-Fatigue	Δ	Pre-Fatigue	Post-Fatigue	Δ
PAH (g) ^§^	1.44 (0.09)	1.36 (0.08)	−0.085 (0.179)	1.36 (0.07)	1.27 (0.1)	−0.087 (0.168)
RAH (g) ^§^	1.86 (0.14)	2.05 (0.11)	0.167 (0.495)	1.87 (0.11)	1.85 (0.1)	−0.109 (0.454)
PAT (g) ^§^	5.71 (0.45)	5.38 (0.37)	−0.321 (1.267)	5.73 (0.59)	5.91 (0.67)	0.146 (1.256)
RAT (g) ^§^	7.24 (0.53)	7.01 (0.45)	−0.237 (1.416)	7.23 (0.62)	7.37 (0.74)	0.096 (1.296)
MIA (%)	73.71 (1.53)	73.77 (2.02)	−0.055 (5.900)	73.89 (2.13)	75.86 (2.35)	1.972 (5.614)

^§^: Non-parametric variables (Wilcoxon Test), Δ: Delta changes, PAH: Peak Acceleration Head, RAH: Ratio of Acceleration Head, PAT: Peak Acceleration Tibia, RAT: Ratio of Acceleration Tibia, MIA: Magnitude of Impact Absorption.

**Table 2:** Results (mean and standard deviation) of the stride frequency and stride length, pre- and post-fatigue.

	Peripheral Fatigue			Central Fatigue		
	Pre-Fatigue	Post-Fatigue	Δ	Pre-Fatigue	Post-Fatigue	Δ
Stride Frequency (Hz)	177.18 (2.48)	176.65 (2.47)	−0.526 (0.959)	176.39 (2.34)	174.89 (2.14)	−1.503 (1.407)
Stride Length (m)	2.63 (0.145)	2.63 (0.145)	0.006 (0.054)	2.65 (0.143)	2.68 (0.133)	−0.121 (0.603)

Δ: Delta changes, Hz: Stride per second.

**Table 3:** Results (mean and standard deviation) of the impacts in the frequency-domain analysis, pre- and post-fatigue.

	Peripheral Fatigue			Central Fatigue		
	Pre-Fatigue	Post-Fatigue	Δ	Pre-Fatigue	Post-Fatigue	Δ
MHSM_low_ (g^2^/Hz)	0.19 (0.01)	0.18 (0.01)	−0.006 (0.025)	0.18 (0.01)	0.17 (0.01)	−0.017 (0.040)
MTSM_low_ (g^2^/Hz) ^§^	0.14 (0.02)	0.14 (0.01)	−0.008 (0.028)	0.13 (0.01)	0.13 (0.01)	0.004 (0.019)
THSM_low_ (g^2^/Hz)	1.76 (0.13)	1.77 (0.14)	0.018 (0.246)	1.75 (0.13)	1.63 (0.14)	−0.115 (0.362)
TTSM_low_ (g^2^/Hz) ^§^	4.33 (0.53)	4.15 (0.41)	−0.166 (0.638)	4.14 (0.45)	4.20 (0.37)	0.896 (0.474)
MHSM_high_ (g^2^/Hz) ^§^	0.01 (0.00)	0.01 (0.00)	0.000 (0.003)	0.01 (0.00)	0.01 (0.00)	0.000 (0.003)
MTSM_high_ (g^2^/Hz)	0.06 (0.01)	0.06 (0.01)	0.001 (0.013)	0.06 (0.01)	0.08 (0.01) ^††^	0.017 (0.022) *
THSM_high_ (g^2^/Hz) ^§^	0.44 (0.05)	0.39 (0.04)	−0.063 (0.104)	0.40 (0.04)	0.41 (0.05)	0.011 (0.192)
TTSM_high_ (g^2^/Hz) ^§^	3.88 (0.48)	3.80 (0.46)	−0.086 (0.899)	3.56 (0.36)	4.37 (0.47) ^††^	0.811 (1.267) *
ATT_low_ (dB) ^§^	−49.29 (17.61)	−50.75 (15.31)	−0.04 (6.66)	−54.73 (15.81)	−59.25 (16.12) ^†,‡‡^	6.85 (12.37) **
ATT_high_ (dB)	−116.44 (39.80)	−121.86 (34.80)	7.58 (19.41)	−128.40 (14.95)	−147.41 (11.98) ^††,‡‡^	25.94 (8.90) *

^§^: Non-parametric variables (Wilcoxon Test), SD: Standard Deviation, Δ: Delta changes, Low: Lower Frequency Range, High: Higher Frequency Range, MTSM: Maximum Tibial Signal Magnitude, TTSM: Total Tibial Signal Magnitude, MHSM: Maximum Head Signal Magnitude, THSM: Total Head Signal Magnitude, ATT: Shock Attenuation, ^†^: *p* < 0.05 central pre-fatigue vs. central post-fatigue, ^††^: *p* < 0.01 central pre-fatigue vs. central post-fatigue, ^‡‡^: *p* < 0.01 peripheral post-fatigue vs. central post-fatigue, *: *p* < 0.05 Δ peripheral fatigue vs. Δ central fatigue, **: *p* < 0.01 Δ peripheral fatigue vs. Δ central fatigue.

## Data Availability

The dataset generated and analyzed during the current study are available from the corresponding author on reasonable request.
